# Regular Physical Activities and Related Factors among Middle-Aged and Older Adults in Jinan, China: A Cross-Sectional Study

**DOI:** 10.3390/ijerph181910362

**Published:** 2021-10-01

**Authors:** Shukang Wang, Wei Ma, Shu-Mei Wang, Xiangren Yi

**Affiliations:** 1 Department of Biostatistics, School of Public Health, Cheeloo College of Medicine, Shandong University, 44, Wenhuaxi Street, Jinan 250012, China; wsk2001@sdu.edu.cn; 2 Institute for Medical Dataology, Shandong University, 12550, Erhuandong Street, Jinan 250002, China; 3 Department of Epidemiology, School of Public Health, Cheeloo College of Medicine, Shandong University, 44, Wenhuaxi Street, Jinan 250012, China; weima@sdu.edu.cn (W.M.); wshm@sdu.edu.cn (S.-M.W.); 4 Department of Sport and Health, The College of Physical Education, Shandong University, 17923, Jingshi Street, Jinan 250061, China

**Keywords:** China, cross-sectional study, regular physical activities, related factors

## Abstract

The objective of this study was to investigate the prevalence of regular physical activity (RPA) among middle-aged and older adults in urban communities in Jinan, China, and to identify the factors related to RPA. A cross-sectional survey was conducted among middle-aged and elderly urban residents. A total of 1406 participants were included in the final data analysis. The results of the four models consistently showed that the relevant factors of RPA were educational level, previously diagnosed hypertension (PDH) and depression. In terms of educational level, compared with illiteracy, from the first model to the fourth model, the odds ratios (ORs) and 95% confidence intervals (CIs) of senior middle school were 2.072 (1.418, 3.026), 2.072 (1.418, 3.026), 1.905 (1.289, 2.816) and 1.926 (1.302, 2.848), respectively, and the ORs and 95% CIs of college or above were 2.364 (1.462, 3.823), 2.364 (1.462, 3.823), 2.001 (1.208, 3.312) and 2.054 (1.239, 3.405). In terms of PDH, compared with those with PDH, from the first model to the fourth model, ORs and 95% CIs of non-PDH were 1.259 (1.003, 1.580), 1.259 (1.003, 1.580), 1.263 (1.006, 1.585) and 1.261 (1.004, 1.584), respectively. For depression, compared with those without depression, also from the first model to the fourth model, ORs and 95% CIs of depression were 0.702 (0.517, 0.951), 0.702 (0.517, 0.951), 0.722 (0.532, 0.981) and 0.719 (0.529, 0.977), respectively. In conclusion, the results of this study showed that participation in RPA among middle-aged and older adults in Jinan urban communities was significantly associated with education level, PDH and depression.

## 1. Introduction

Population aging is a global social problem, and a momentous response strategy is healthy aging. Regular physical activity (RPA) plays an important role in delaying the functional decline of adults and reducing the risk of major chronic diseases, such as hypertension, type 2 diabetes mellitus (T2DM), certain cancers, depression and dementia, and mortality, and so it is a crucial way for healthy aging [1,2,3,4,5]. Although the effect of RPA in promoting the physical and mental health of older adults is recognized, there are still many middle-aged and older adults not meeting the recommended standards for physical activities [6]. The 2015 Global Health Survey from the World Health Organization (WHO) showed that about 30% of people aged 70 to 79 and 50% of people aged 80 and over did not meet the recommended physical activity standards [7]. The lack of RPA in older adults is a problem worthy of attention.

Many studies showed that demographic factors, environmental factors, social support, lifestyle factors and health conditions are related to RPA of older adults [8,9,10]. Environmental factors mainly include the built environment, traffic safety, parks and open space, and results of studies from some countries including China agree that a good environment could help people participate in RPA [11,12]. As for social support, similarly, many studies, including Chinese studies, reached the same conclusion that social support could promote physical activities in older adults [13,14,15,16].

However, although many studies showed that RPA was related to demographic factors such as age, gender, educational level, marital status and income status, the results were not consistent [17,18], perhaps due to differences in countries or regions [19]. A study from six Asia-Pacific countries showed that physical activities were positively correlated with age in China, negatively correlated with age in men from Fiji and Nauru, and had no correlation with age in other countries [20]. A study from Iran showed that the physical activity levels of the older adults were negatively correlated with age and positively correlated with educational level and that women or unmarried had lower levels of physical activity [17]. A study from Shenzhen, China, revealed a negative association between educational level and physical activity, and individuals with higher education levels were more likely to report less physical activity [21]. Moreover, some studies showed that RPA was associated with lifestyle factors such as smoking [22,23], alcohol drinking [24,25] and body mass index (BMI) [26,27], but the results remain inconsistent. Further, most studies on the relationship between physical activities and health conditions showed that physical activities could improve certain chronic diseases for older adults. The prescription of RPA tailored for T2DM patients had been shown to be highly effective for glycemic control [28]. Brisk walking could reduce the magnitude of blood pressure rise during exercise of different intensities, thereby reducing the risk of acute cardiovascular incidents in older patients with essential hypertension [29]. RPA might also lower lipid levels to reduce the risk of cardiovascular disease [30,31]. The above were all studies on the effects of RPA on chronic diseases. In turn, few studies with RPA as the outcome variable have focused on the effects of various chronic disease conditions on the RPA of older adults, especially in China.

Therefore, we investigated the prevalence of RPA and analyzed its associated factors for middle-aged and older adults in Jinan urban communities.

## 2. Materials and Methods

### 2.1. Study Population

A cross-sectional survey was conducted among middle-aged and older urban residents from 2011 to 2012. These residents were from several randomly selected neighborhoods in 6 districts of Jinan City, China. Using the stratified random sampling method, a total of 12 neighborhoods were randomly selected from the six districts of Jinan City. In this survey, a total of 3309 subjects aged 45 years or older completed the questionnaire, and their mean age was 64.11 years. In this study, the inclusion criteria for the subjects were as follows: (1) aged 45 years or older, (2) able to answer the questions in the questionnaire and (3) have lived in the selected communities for more than half a year in the past year. In total, 1903 participants were excluded because of the lack of information on certain variables such as marital status, educational level, income status, smoking, alcohol drinking, BMI, triglyceride (TG), total cholesterol (TC), previously diagnosed hypertension (PDH), previously diagnosed type 2 diabetes mellitus (PDM), depression and RPA. A total of 1406 participants were included in the final data analysis. This study was approved by the Ethics Committee of the School of Public Health, Shandong University, and written informed consent was obtained from all participants (Code No. 20110301).

### 2.2. Investigation and Measurements

All participants were surveyed at a designated location near their communities. During the survey process, with a self-designed standardized questionnaire and face-to-face inquiries, the variable information of the subjects was collected by trained interviewers. Participants were asked about their age, gender, marital status, education level, income status, smoking status, alcohol drinking status, whether they were PDH and whether they were PDM. Moreover, three questions related to physical activity, namely frequency, time and symptoms after physical activity, were included in the questionnaire. The details of categorical variables are presented in Table 1. Using Self-Rating Depression Scale questionnaires (SDS), which is a 20-item self-report measure of the symptoms of depression, each subject received a depression score [32]. Each participant was told to score each item, which contained four levels (never or few times = 1, sometimes = 2, often = 3, almost always or always = 4). The total score of 20 items is between 0 and 80. Depression index was equal to the individual’s total score from 20 items divided by 80. After an overnight fast, all participants went through a standard physical examination; their height and weight were measured without their shoes, heavy clothing or belts by trained nurses. The height and weight values were averaged by two surveyors through different instruments. BMI was calculated as weight (kg) divided by square of height (m). Fasting blood samples were taken at the survey site by experienced nurses and then sent to the designated hospital and tested within two hours. Laboratory testing indicators included TG and TC required in this study.

### 2.3. Definitions of PDH, PDM, Depression and RPA

PDH was defined as hypertension previously diagnosed by doctors. PDM was defined as T2DM previously diagnosed by doctors. The depression indexes with 0–0.49 and 0.50–1.00 represent no depression and depression, respectively [33].

The definition of RPA was based on the three questions in the questionnaire, including the frequency of physical activities per week, the duration of each activity and the symptoms after each activity. The options for these three questions are presented in Table 1. We defined the intensity of physical activities based on the symptoms after each activity; moderate-intensity physical activities were defined as breathing and heartbeat speeding up slightly and slight sweating. According to physical activity guidelines for older adults and the objective of this study [34], RPA needed to meet the following three conditions at the same time: (1) 3 times or more physical activities per week, (2) the duration of each physical activity being 30 min and above and (3) moderate-intensity and above physical activity each time.

According to Chinese standards [35], BMI was stratified as follows: BMI < 18.5 kg/m^2^ means underweight, 18.5 kg/m^2^ ≤ BMI < 23.9 kg/m^2^ means normal weight, 24 kg/m^2^ ≤ BMI < 27.9 kg/m^2^ means overweight and BMI ≥ 28 kg/m^2^ means obesity. Hypertriglyceridemia was defined as TG ≥ 2.26 mmol/L. Hypercholesterolemia was defined as TC ≥ 6.22 mmol/L [36].

### 2.4. Statistical Analysis

The percentage of each category within various characteristics was calculated by gender. All characteristics of the RPA group and the non-RPA group were statistically described. Mean and standard deviation (mean ± s.d.) were used to describe the numerical variables, and percentages were used to describe the categorical variables. We used *t*-test to compare the distribution in age, BMI, TG and TC between the RPA group and the non-RPA group. Chi-square test was used to compare nominal variables. Binary logistic regression was used to analyze the related factors of RPA behavior. Four logistic regression models were established according to the different variables contained in the models. Education level, PDH and depression were included in the first model. Compared to the first model, age and gender were added to the second model. Educational level, PDH, depression, age, gender, marital status, smoking and alcohol drinking were included in the third model. Compared with the third model, PDM, BMI, TG and TC were added to the fourth model. All statistical analyses were performed with SPSS23.0 software (IBM Inc., Armonk, NY, USA). The statistical significance was defined as a *p* < 0.05.

## 3. Results

In this study, there were 1406 middle-aged and older participants, 446 men and 960 women. The prevalence of RPA was 44% among middle-aged and older adults in Jinan, China.

For male, female and all participants, Table 1 shows the numbers and percentages of participants in different categories of various characteristics, namely age, marital status, educational level, income status, smoking, alcohol drinking, BMI, hypertriglyceridemia, hypercholesterolemia, PDH, PDM, depression, RPA, the frequency of physical activities, the duration of each physical activity and the symptoms after physical activities.

Table 2 shows the comparison of age, BMI, TG and TC between the RPA group and the non-RPA group, and only the difference in age was statistically significant (*p* = 0.002). The comparison results of the prevalence of RPA among different types of groups of various characteristics such as gender, marital status, educational level, income status, smoking, alcohol drinking, PDH, PDM and depression are also presented in Table 2. There were significant differences in the prevalence of RPA between groups of different marital status (*p* = 0.003), education level (*p* < 0.001) or income status (*p* < 0.001). Similarly, there was a significant difference in the prevalence of RPA between the PDH group and the non-PDH group (*p* = 0.007), as was found for depression (*p* = 0.003).

Table 3 shows the relationship between RPA and statistically significant independent variables in the four models. The number of independent variables gradually increased from the first model to the fourth model, and the variables included in each model are listed below in Table 3. The results in the four models consistently showed that the factors that were significantly related to RPA were educational level, PDH and depression. For example, in the fourth model, with adjustment for age, gender, marital status, income status, smoking, alcohol drinking, PDM, BMI, TG and TC, for the variable of education level, the odds ratios (ORs) and 95% confidence intervals (CIs) of senior middle school and college or above compared with illiteracy were 1.926 (1.302, 2.848) and 2.054 (1.239, 3.405), respectively; the OR and 95% CI of non-PDH were 1.261 (1.004, 1.584) compared with PDH, and the OR and 95% CI of depression were 0.719 (0.529, 0.977) compared with nondepression. It can be seen that a higher level of education or no PDH may increase RPA, while depression may reduce RPA.

## 4. Discussion

### 4.1. Summary of the Results of This Study

This study found that more than half of middle-aged and older adults in Jinan did not engage in RPA. The results showed that educational level, PDH and depression were significantly related to RPA participation, adjusting for some demographic factors, lifestyle factors and chronic disease conditions. A further key finding was that the participation in RPA was negatively correlated with PDH and depression but was not correlated with PDM. In other words, the participation of older adults in RPA may be reduced due to PDH and depression.

### 4.2. Comparison of the Prevalence of RPA

In this study, the prevalence of RPA was 44% among middle-aged and older adults in Jinan, China. The WHO reported that the proportion of younger Chinese older adults (50–59 years old) who participated in RPA was 72.1%, while the proportion of older adults aged 70 or older was 55.9% in 2007–2010, and in urban areas, the prevalence of RPA of older adults ranged from 49.4% in Shandong to 80.6% in Guangdong [37]. RPA in the above study was defined as more than 150 min of moderate or vigorous physical activities per week according to the WHO-recommended cut-off point [38], while RPA was defined as 3 times a week or more and 30 min or more of moderate or vigorous physical activities each time in our study. In our study, the standard of RPA was lower than that of the WHO in order to analyze the possible effect of chronic disease conditions on RPA among older adults [34]. It can be seen that the RPA level of older adults in Jinan was relatively low.

### 4.3. The Relationship between RPA and Demographic Factors

Among the demographic factors in this study, only educational level was consistently found to be related to RPA from the first model to the fourth model, and compared with illiteracy, participants with an educational level of senior middle school and college or above exhibited increased RPA. Similar to the results of this study, most previous studies have also shown that older adults with a higher educational level were more likely to have higher levels of RPA [39]. Perhaps a higher educational level of older adults led to the better cognition of health to recognize the health benefits of RPA [40]. This also suggests that health education for older adults should be strengthened, especially for the older adults with low levels of education, so that they can realize the health benefits of RPA.

The results of some studies on the associations between RPA and other demographic factors such as age, gender, marital status and income status were inconsistent in different countries [41]. For example, one study showed no significant association between age and physical activity level [20], while another study showed that physical activities of older adults had a negative relationship with age [42]. As for studies on gender, in 53 studies included in a review, men were more active than women in 27 studies and less active in 7 studies, and 19 studies showed that gender was not related to RPA [43]. Similarly, the results on marital status or income level were also inconsistent [12,44,45]. Our results showed that RPA participation of older adults in urban communities in Jinan was not related to age, gender, marital status, and income level. In fact, the key is to identify the characteristics related to RPA of different elderly groups in different countries and regions, so as to adopt effective measures to promote the participation in RPA of older adults in a targeted manner.

### 4.4. The Relationship between RPA and Chronic Disease Conditions

Regarding the relationship between RPA and hypertension, most previous studies focused on the effect of physical activities on hypertension. The results of randomized controlled trials (RCTs) showed RPA could reduce the blood pressure of patients with hypertension [46]. The results of the decision tree showed that vigorous physical activity seemed to be more important than moderate and light activity to induce beneficial effects on the prevention of hypertension [47]. However, in turn, few studies used RPA participation as an outcome variable to analyze the possible effects of PDH on RPA. The results of this study showed that the RPA participation of older adults in the PDH group was lower than that in the non-PDH group. There may be two reasons for this. On the one hand, hypertension and its secondary cardiovascular and cerebrovascular diseases restricted the participation in RPA. For example, elderly patients with heart failure (HF) had decreased physical function due to decreased cardiorespiratory health and muscle strength, thereby reducing participation in physical activities [48]. On the other hand, compared with older adults without PDH, people with PDH were more likely to have lower self-efficacy [49], which reduced physical activities [50]. In addition, health intervention studies also supported our results, showing that the health conditions of hypertensive patients were also the main reason why these patients did not participate in physical activity programs [51]. Different from the above results, a cross-sectional study reported that moderate-to-vigorous physical activity (MVPA) of older Germans was not related to hypertension. This difference from our study may be due to the definition of hypertension and the study only including individuals aged 48–68 [52]. Maybe chronic disease conditions of the younger elderly (aged < 70 years) did not affect their participation in RPA [53]. Different from the definition of hypertension in the German study, PDH in our study meant previously diagnosed hypertension; this negative relationship between PDH and RPA might show a greater psychological burden on older adults [54]. In other words, older adults with PDH may worry about accidents and physical discomfort due to physical activities [55]. Therefore, it is recommended to pay attention to RPA participation of older adults with PDH, conduct health education for them, and develop specific RPA plans for them.

Increasing evidence generally indicates that RPA is beneficial in preventing and improving depression among older adults [56,57]. Similar to studies on the relationship between hypertension and RPA, few studies have focused on the effect of depression on RPA in older adults. In this study, participation in RPA was used as the dependent variable, and the results showed a negative relationship between depression and RPA. Some studies elaborated on the same viewpoints as this study. Depression might reduce participation in RPA through lower motivation and willpower [58,59]. For example, older adults who did not participate in RPA showed higher depression compared with those who participated in RPA [60]. Actually, individuals with cognitive impairment have less autonomy, as well as less ability to perceive the benefits of RPA [61], and so preventing depression was a key factor in promoting RPA [62]. Prospective studies showed that the association between RPA and depressive symptoms appeared to be bidirectional [63,64] due to the relationship between physical function and physical activities being bidirectional [65]. Elderly people who were clinically diagnosed with depression took part less in RPA [66], which aggravated the symptoms of depression [67]. Therefore, RPA of older adults with depression should be focused on, so that they could enter a virtuous circle as soon as possible, that is, from the increase in RPA to the reduction in depression, and then increase their participation in RPA.

In addition, our findings showed that RPA was not associated with PDM, BMI, TG and TC. Some cross-sectional studies showed that RPA was negatively correlated with BMI, TG, TC and T2DM in older adults [68,69,70]. The above-mentioned noncorrelation or negative correlation between RPA and T2DM or metabolic indicators shown in the cross-sectional studies indicated that older adults without RPA had not changed their lifestyles due to these chronic disease conditions.

### 4.5. Limitations of This Study

Our investigation had several limitations. First, our study had a cross-sectional design, which could be used to explore the related factors of RPA but could not be used to explain causation. Further intervention experiments are important to determine the effects of chronic disease conditions on RPA in older adults. Second, more than half of the participants in this study did not take part in the physical examination, and data on some health conditions, cognitive abilities, neuropsychological assessments, eating and drinking disorders and physical activity measurement based on standardized questionnaires were lacking. These may have created some bias. Third, RPA for middle-aged and older adults is of great significance to the realization of healthy aging. Although the inclusion criteria for aging concern subjects aged 60 years or older, younger participants were considered in this study.

## 5. Conclusions

In this study, the prevalence of RPA among middle-aged and older adults was 44%, which was relatively low. This study analyzed the factors related to RPA participation of middle-aged and older adults in Jinan urban communities, in China. The results showed that the participation in RPA was significantly associated with education level, PDH and depression but was not associated with age, gender, marital status, income status, smoking, alcohol drinking, PDM, BMI, TG and TC.

## Figures and Tables

**Table 1:** Summary statistics of characteristics according to gender (*n* (%)).

Characteristic	*n* (%)		
	Males (*n* = 446)	Females (*n* = 960)	Total (*n* = 1406)
Age (years)			
45–54	60 (13.5)	202 (21.1)	262 (18.6)
55–64	175 (39.2)	364 (37.9)	539 (38.3)
65–74	133 (29.8)	242 (25.2)	375 (26.7)
≥75	78 (17.5)	152 (15.8)	230 (16.4)
Marital status			
Married	411 (92.2)	733 (76.4)	1144 (81.4)
Not married	35 (7.8)	227 (23.6)	262 (18.6)
Educational level			
Illiteracy	7 (1.6)	158 (16.5)	165 (11.7)
Elementary school	62 (13.9)	187 (19.5)	249 (17.7)
Junior middle school	112 (25.1)	244 (25.4)	356 (25.3)
Senior middle school	182 (40.8)	322 (33.5)	504 (35.9)
College or above	83 (18.6)	49 (5.1)	132 (9.4)
Income status (CNY)			
<1000	61 (13.7)	170 (17.7)	231 (16.4)
1000–2000	138 (30.9)	549 (57.2)	687 (48.9)
≥2000	247 (55.4)	241 (25.1)	488 (34.7)
Smoking			
Yes	172 (38.6)	32 (3.3)	204 (14.5)
No	274 (61.4)	928 (96.7)	1202 (85.5)
Alcohol drinking			
Yes	145 (32.5)	21 (2.2)	166 (11.8)
No	301 (67.5)	939 (97.8)	1240 (88.2)
BMI			
<18.5	6 (1.3)	24 (2.5)	30 (2.1)
18.5–23.9	130 (29.1)	311 (32.4)	441 (31.4)
24.0–27.9	234 (52.5)	417 (43.4)	651 (46.3)
≥28.0	76 (17.1)	208 (21.7)	284 (20.2)
Hypertriglyceridemia (mmol/L)			
<2.26	386 (86.5)	823 (85.7)	1209 (86.0)
≥2.26	60 (13.5)	137 (14.3)	197 (14.0)
Hypercholesterolemia (mmol/L)			
<6.22	419 (93.9)	795 (82.8)	1214 (86.3)
≥6.22	27 (6.1)	165 (17.2)	192 (13.7)
PDH			
Yes	157 (35.2)	336 (35.0)	493 (35.1)
No	289 (64.8)	624 (65.0)	913 (64.9)
PDM			
Yes	66 (14.8)	103 (10.7)	169 (12.0)
No	380 (85.2)	857 (89.3)	1237 (88.0)
Depression			
Yes	56 (12.6)	167 (17.4)	223 (15.9)
No	390 (87.4)	793 (82.6)	1183 (84.1)
RPA			
Yes	208 (46.6)	411 (42.8)	619 (44.0)
No	238 (53.4)	549 (57.2)	787 (56.0)
The frequency of PA			
<1 time a week	39 (8.7)	97 (10.1)	136 (9.7)
1–2 times a week	36 (8.1)	58 (6.0)	94 (6.7)
3–4 times a week	46 (10.3)	98 (10.2)	144 (10.2)
5–7 times a week	213 (47.8)	471 (49.1)	684 (48.6)
≥8 times a week	112 (25.1)	236 (24.6)	348 (24.8)
The duration of each PA			
<15 min	36 (8.1)	89 (9.3)	125 (8.9)
15–30 min	36 (8.1)	70 (7.3)	106 (7.5)
30–60 min	113 (25.3)	272 (28.3)	385 (27.4)
60–90 min	155 (34.7)	338 (35.2)	493 (35.1)
≥90 min	106 (23.8)	191 (19.9)	297 (21.1)
The symptoms after PA			
LCBH	199 (44.6)	473 (49.3)	672 (47.8)
BHSUS	210 (47.1)	401 (41.8)	611 (43.5)
FBH	32 (7.2)	81 (8.4)	113 (8.0)
RBH	5 (1.1)	5 (0.5)	10 (0.7)

BMI, body mass index; PDH, previously diagnosed hypertension; PDM, previously diagnosed type 2 diabetes mellitus; RPA, regular physical activity; PA, physical activity; LCBH, little change in breathing and heartbeat, usually no sweating; BHSUS, breathing and heartbeat sped up slightly, sweating slightly; FBH, fast breathing and heartbeat, sweating more; RBH, rapid breathing and heartbeat, sweating profusely; CNY, Chinese yuan.

**Table 2:** Summary statistics according to RPA and comparisons of different types of groups of various characteristics (means ± s.d.).

Characteristic	RPA (*n* = 619)	No RPA (*n* = 787)	*p*
Age (years)	62.91 ± 8.80	64.46 ± 9.86	0.002
BMI (kg/m^2^)	25.33 ± 3.35	25.42 ± 3.63	0.624
TG (mmol/L)	1.46 ± 0.94	1.51 ± 0.92	0.411
TC (mmol/L)	5.31 ± 1.14	5.20 ± 0.91	0.067
Gender, *n* (%)			0.179
Male	208 (46.6)	238 (53.4)	
Female	411 (42.8)	549 (57.2)	
Marital status, *n* (%)			0.003
Married	525 (45.9)	619 (54.1)	
Not married	94 (35.9)	168 (64.1)	
Educational level, *n* (%)			<0.001
Illiteracy	51 (30.9)	114 (69.1)	
Elementary school	100 (40.2)	149 (59.8)	
Junior middle school	143 (40.2)	213 (59.8)	
Senior middle school	254 (50.4)	250 (49.6)	
College or above	71 (53.8)	61 (46.2)	
Income status, *n* (%)			<0.001
<1000	82 (35.5)	149 (64.5)	
1000–2000	291 (42.4)	396 (57.6)	
≥2000	246 (50.4)	242 (49.6)	
Smoking, *n* (%)			0.375
Yes	84 (41.2)	120 (58.8)	
No	535 (44.5)	667 (55.5)	
Alcohol drinking, *n* (%)			0.627
Yes	76 (45.8)	90 (54.2)	
No	543 (43.8)	697 (56.2)	
PDH, *n* (%)			0.007
Yes	193 (39.1)	300 (60.9)	
No	426 (46.7)	487 (53.3)	
PDM, *n* (%)			0.691
Yes	72 (42.6)	97 (57.4)	
No	547 (44.2)	690 (55.8)	
Depression, *n* (%)			0.003
Yes	78 (35.0)	145 (65.0)	
No	541 (45.7)	642 (54.3)	

BMI, body mass index; TG, triglyceride; TC, total cholesterol; PDH, previously diagnosed hypertension; PDM, previously diagnosed type 2 diabetes mellitus.

**Table 3:** ORs and their 95% CIs of the related factors of RPA from logistic models.

Variables	Model ^1^	Model ^2^	Model ^3^	Model ^4^
Educational level				
Illiteracy	ref	ref	ref	ref
Elementary school	1.415 (0.930, 2.153)	1.415 (0.930, 2.153)	1.365 (0.894, 2.085)	1.392 (0.911, 2.128)
Junior middle school	1.389 (0.934, 2.067)	1.389 (0.934, 2.067)	1.337 (0.894, 1.998)	1.367 (0.913, 2.045)
Senior middle school	2.072 (1.418, 3.026) *	2.072 (1.418, 3.026) *	1.905 (1.289, 2.816) *	1.926 (1.302, 2.848) *
College or above	2.364 (1.462, 3.823) *	2.364 (1.462, 3.823) *	2.001 (1.208, 3.312) *	2.054 (1.239, 3.405) *
PDH				
Yes	ref	ref	ref	ref
No	1.259 (1.003, 1.580) *	1.259 (1.003, 1.580) *	1.263 (1.006, 1.585) *	1.261 (1.004, 1.584) *
Depression				
No	ref	ref	ref	ref
Yes	0.702 (0.517, 0.951) *	0.702 (0.517, 0.951) *	0.722 (0.532, 0.981) *	0.719 (0.529, 0.977) *
Income status, *n* (%)				
<1000			ref	ref
1000–2000			1.146 (0.831, 1.579)	1.126 (0.816, 1.553)
≥2000			1.417 (1.002, 2.003) *	1.403 (0.992, 1.985)

PDH, previously diagnosed hypertension; Model ^1^: included educational level, PDH and depression; Model ^2^: included educational level, PDH, depression, gender and age; Model ^3^: included educational level, PDH, depression, age, gender, marital status, income status, smoking and alcohol drinking; Model ^4^: included educational level, PDH, depression, age, gender, marital status, income status, smoking, alcohol drinking, PDM, BMI, TG and TC; * *p* < 0.05.

## Data Availability

The data presented in this study are available on request from the corresponding author.
